# 

**Published:** 1934

**Authors:** 


PUBLISHED UNDER THE AUSPICES OF

THE BRISTOL MEDICO-CHIRURGICAL SOCIETY.

*Editor:* J. A. NIXON, C.M.G., M.D., F.R.C.P.

WITH WHOM ARE ASSOCIATED

E. W. HEY GROVES, D.Sc., M.S., F.R.C.S.
A. E. ILES, O.B.E., M.B., B.S., F.R.C.S.
A. RENDLE SHORT, M.D., B.S., B.Sc., F.R.C.S.

E. WATSON-WILLIAMS, M.C., Ch.M., F.R.C.S., *Assistant Editor.*

*Editorial Secretary:* A. L. FLEMMING, M.B., Ch.B.

BRISTOL: J. W. ARROWSMITH LTD., 11 QUAY STREET.
London: J. W. Arrowsmith (London) Ltd., 8 Endsleigh Gardens, W.C.1.

Price Three Shillings net.

